# Identification and Molecular Characterization of a Pellino Protein in Kuruma Prawn (*Marsupenaeus Japonicus*) in Response to White Spot Syndrome Virus and *Vibrio Parahaemolyticus* Infection

**DOI:** 10.3390/ijms21041243

**Published:** 2020-02-13

**Authors:** Heqian Zhang, Wenzhi Cheng, Jinbin Zheng, Panpan Wang, Qinghui Liu, Zhen Li, Tianyi Shi, Yijian Zhou, Yong Mao, Xiangyong Yu

**Affiliations:** 1 Joint Laboratory of Guangdong Province and Hong Kong Regions on Marine Bioresource Conservation and Exploitation, College of Marine Sciences, South China Agricultural University, Guangzhou 510642, China; 20171067005@stu.scau.edu.cn (H.Z.); 20172067002@stu.scau.edu.cn (Q.L.); 20183140004@stu.scau.edu.cn (Z.L.); 2 State Key Laboratory of Marine Environmental Science, College of Ocean and Earth Sciences, Xiamen University, Xiamen 361102, China; 22320170154939@stu.xmu.edu.cn (W.C.); zhengjinbin@nbu.edu.cn (J.Z.); 22320160153955@stu.xmu.edu.cn (P.W.); 22320181152137@stu.xmu.edu.cn (T.S.); 22320171150846@stu.xmu.edu.cn (Y.Z.); 3 Fujian Key Laboratory of Genetics and Breeding of Marine Organisms, Xiamen University, Xiamen 361102, China

**Keywords:** MjPellino, WSSV, *Vibrio parahaemolyticus*, VP26, protein-protein docking, immune response, *Marsupenaeus japonicus*

## Abstract

Kuruma prawn, *Marsupenaeus japonicus*, has the third largest annual yield among shrimp species with vital economic significance in China. White spot syndrome virus (WSSV) is a great threat to the global shrimp farming industry and results in high mortality. Pellino, a highly conserved E3 ubiquitin ligase, has been found to be an important modulator of the Toll-like receptor (TLR) signaling pathways that participate in the innate immune response and ubiquitination. In the present study, the Pellino gene from *Marsupenaeus japonicus* was identified. A qRT-PCR assay showed the presence of MjPellino in all the tested tissues and revealed that the transcript level of this gene was significantly upregulated in both the gills and hemocytes after challenge with WSSV and *Vibrio parahaemolyticus*. The function of MjPellino was further verified at the protein level. The results of the three-dimensional modeling and protein–protein docking analyses and a GST pull-down assay revealed that the MjPellino protein was able to bind to the WSSV envelope protein VP26. In addition, the knockdown of MjPellino in vivo significantly decreased the expression of MjAMPs. These results suggest that MjPellino might play an important role in the immune response of kuruma prawn.

## 1. Introduction

Kuruma prawn, *Marsupenaeus japonicus* (Decapoda, Penaeidae, *Marsupenaeus*), is widely distributed in the Indo-Western Pacific region and the East and South China Seas [1]. The global growth rate of economically crucial prawns in aquaculture has exponentially increased among crustacean species [2]. In particular, kuruma shrimp constitutes one of the major shrimp species with high economic value that is mainly cultivated in China. Nevertheless, the rapid growth rate in the production of these shrimp has been followed by outbreaks of various diseases, which have resulted in the current crisis.

*M*. *japonicus* cultivation has been threatened by a wide range of diseases, such as those caused by viruses and bacteria, which are the most dangerous pathogens of shrimp in aquaculture [3]. *Vibrio parahaemolyticus* is a common pathogenic bacterium which can cause diseases in marine aquaculture and leads to huge economic damage to the aquaculture industry [4]. Another major pathogen in shrimp aquaculture is the white spot syndrome virus (WSSV), an enveloped, double-stranded DNA virus that causes white spot syndrome (WSS) in its crustacean hosts, is a pernicious pathogen that is responsible for high mortality in cultured prawns (100% cumulative mortality within a few days after infection) and is a major cause for concern in the aquaculture industry [5,6,7]. The unique envelope proteins of WSSV might play a pivotal role in initiating the innate immune response and might play crucial roles in cell targeting, viral entry, assembly, budding, and generation of the host antiviral defense system that makes WSSV such a lethal pathogen [8,9,10,11]. VP19, VP24, VP26, and VP28 are the four most abundance envelope proteins of WSSV [12]. The structural protein family WSS_VP (Pfam accession no. PF12175 includes three major WSSV structural proteins, namely, VP24, VP26 andVP28 [11]. The viral envelope is composed of a network of interactions among the various building blocks [13]. Tang [14] showed that the envelope proteins of WSSV might interact with the host cells to ensure efficient viral infection.

Crustaceans mostly depend on their innate immune system for the elimination of pathogens [15]. The innate immune response is the first line of defense against different types of infections in both vertebrate and invertebrate animals [15]. In crustaceans, the Toll and IMD pathways mediate innate immunity. Toll-like receptors (TLRs) organize early barriers against infectious diseases [16]. However, there still remain many ambiguities and gaps in knowledge regarding the TLRs network, particularly in lower organisms [17]. Pellino, which contains an E3 ubiquitin ligase domain, has been found to be a highly conserved module of the Toll pathway, as initially identified in *Drosophila* [18]. Pellino1, Pellino2 and Pellino3 were characterized in mammals based on their involvement in regulating TLR-mediated innate immune responses [19,20,21,22,23]. The C-terminus of Pellino contains a RING domain, which has E3 ubiquitin ligase activity and catalyzes the polymerization of IRAK1 phosphorylation, whereas the N- terminus contains a forkhead-associated (FHA) domain, which can promote binding to phosphorylated IRAK1 and Pelle [18,24,25]. The available information regarding the Pellino gene in crustaceans is limited. To date, the crustacean Pellino genes in *Litopenaeus vannamei*, *Penaeus monodon* and *Scylla paramamosain* have been included in the GenBank nucleotide sequence database [26]. At the transcript level, overexpression of the *L. vannamei* Pellino gene can increase the activity of promoters with some NF-κB-binding DNA motifs, such as the promoters of WSSV and arthropod antimicrobial peptides (AMPs) [27]. However, no relevant studies have investigated the mechanism through which the Pellino gene participates in the WSSV infection process at the protein translation level, which is of great significance.

However, the available information on the Pellino gene and its foundations and induced expression mode in kuruma prawn is scarce. In the present study, MjPellino was characterized and identified from transcriptomic data obtained from *M. japonicus* and its tissue distributions and transcriptional profiles in response to WSSV and *V. parahaemolyticus* challenges were clarified by the quantitative real-time polymerase chain reaction analysis. Additionally, protein–protein docking assays and a glutathione-S-transferase (GST) pull-down assay were employed to investigate the interactions between MjPellino and four WSSV envelope proteins. The functions of MjPellino in vivo were further investigated using RNA interference (RNAi) strategy. The identification of MjPellino can help us better understand the vital functions of this protein in shrimp innate immunity.

## 2. Results

### 2.1. Characterization of the cDNA Sequence of MjPellino

**Figure 1:**
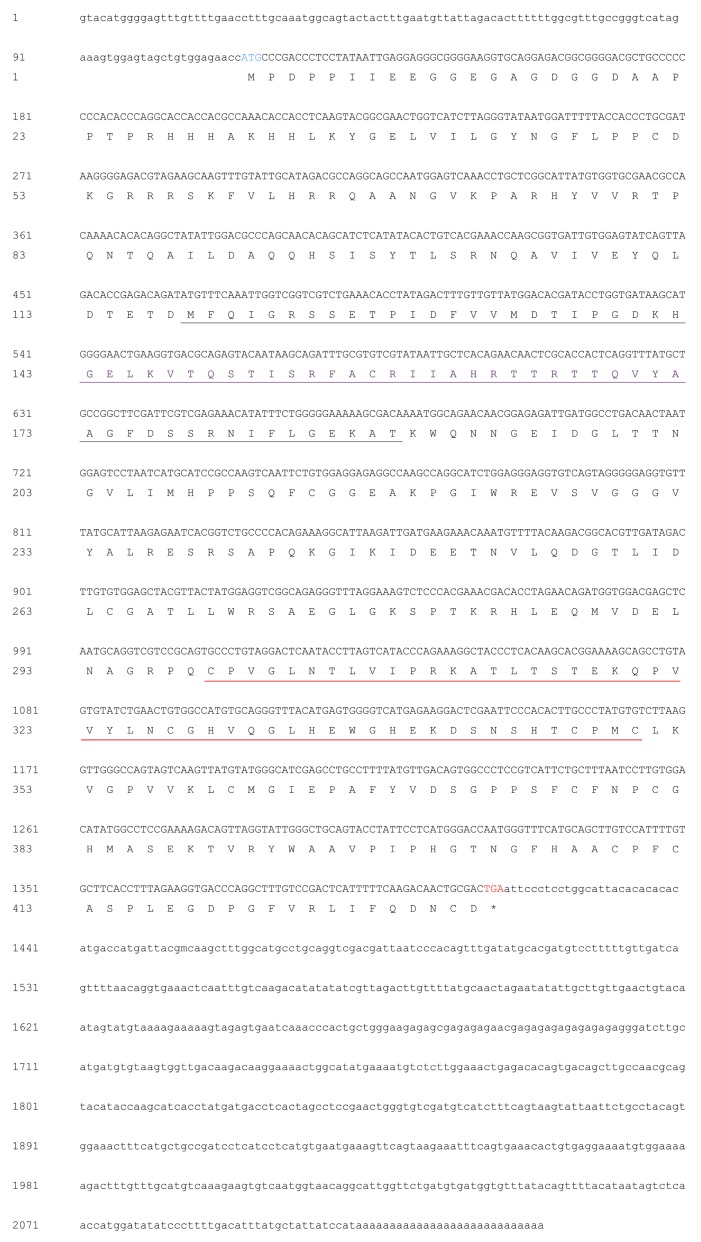
mRNA and deduced amino acid (AA) sequences of MjPellino. The ORF of the nucleotide sequence is shown in uppercase letters, whereas the 5′- and 3′-untranslated region (UTR) sequences are shown in lowercase letters. The initiation codon ATG is highlighted in blue, and the termination codon TGA is highlighted in red. The forkhead-associated (FHA) domain in the N-terminal 118–188 region is underlined in gray, and the RING E3 ubiquitin ligase domain is marked with a red underline.

The MjPellino mRNA sequence is 2139 in length and contains a 114-bp (1-114) 5′-untranslated region (UTR) and a 726-bp (1414–2139) 3′-UTR, and the ORF from positions 115 to 1413 (1299 bp) encode a 432-amino-acid protein with a calculated molecular weight of 47.3 kDa (Figure 1) that belongs to the Pellino family (InterPro No. IPR006800). No signal peptide was detected using the online SignalP-5.0 server [28]. An architectural analysis showed that the MjPellino protein sequence contains an FHA domain in the N-terminal 118–188 region and a CHC2CHC2 RING E3 ubiquitin ligase domain in the C-terminal 299–350 region. The obtained MjPellino sequence has been deposited in NCBI GenBank [26] with the GenBank accession number MN307903.

### 2.2. Multiple-Sequence Alignment and Phylogenetic Analysis

A multiple-sequence alignment of Pellino homologs demonstrated that the AA sequence of MjPellino shared relatively high similarity with the homologous proteins from the other examined species. Notably, Pellino proteins are ubiquitously highly conserved, particularly in different shrimp species (Supplementary Figures S1). The full-length sequences of the MjPellino protein and its homologs in other species were subjected to phylogenetic analysis based on the NJ method using MEGA7.0 software [29]. In the NJ phylogenetic tree, the Chordata and Arthropoda Pellino proteins included in this study were clustered in a subtree that included branches consisting of the *A. queenslandica* (Protozoa) Pellino protein and *C. elegans* (Nematomorpha) Pellino protein. Phylogenetic analysis showed that MjPellino was mostly clustered within the subtree of Arthropoda Pellino proteins (Supplementary Figures S2).

### 2.3. Tissue Distribution of MjPellino

**Figure 2:**
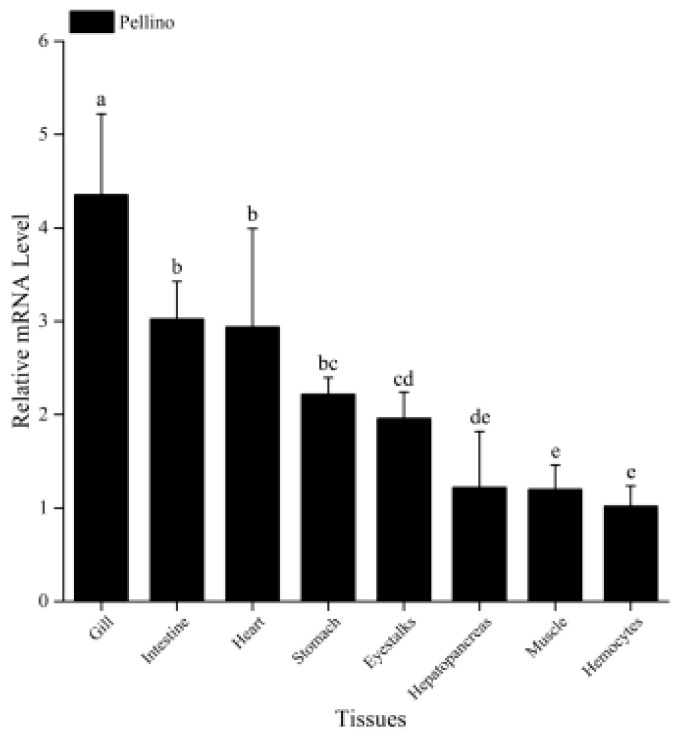
Tissue distribution of MjPellino mRNA expression in healthy *M. japonicus*. The expression levels in the gill, intestine, heart, stomach, eyestalks, hepatopancreas, and muscle were normalized to that in hemocytes. Each bar represents the mean ± SD (*n* = 6). A significant difference between groups (*p* < 0.05, ANOVA) is indicated by different letters (a, b, c, d and e) above the bars.

The qRT-PCR results showed that MjPellino mRNA expression could be detected in all the different tissues tested in this study, including the hemocytes, muscle, hepatopancreas, eyestalks, stomach, heart, intestine, and gill of healthy shrimp. The highest MjPellino mRNA expression was detected in gill tissue, which is one of the most important immunological tissues of *M. japonicus*, followed by the intestine, and the levels in these two tissues were 4.4-fold and 3.0-fold higher, respectively, than that in hemocytes. Moderate expression was detected in most of the examined tissues, including the hepatopancreas (another important immune tissue), heart, stomach, eyestalk, and muscle tissue, and the lowest expression level was found in hemocytes (Figure 2).

### 2.4. MjPellino was Upregulated by WSSV and Vibrio Parahaemolyticus Challenge

**Figure 3:**
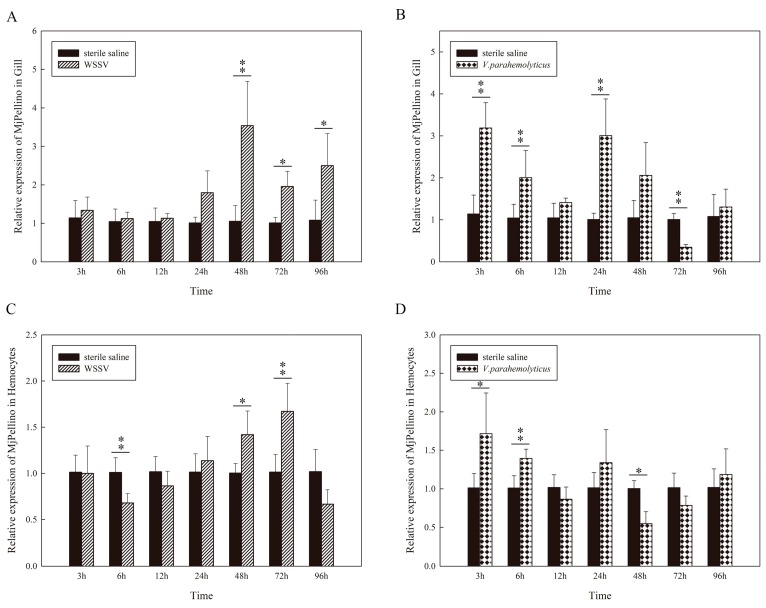
MjPellino mRNA expression levels in the gills and hemocytes of WSSV- and *V. parahaemolyticus*-challenged *M. japonicus*. The temporal expression patterns of MjPellino in the gills (**A**) and hemocytes (**C**) after WSSV challenge and in the gills (**B**) and hemocytes (**D**) after *V. parahaemolyticus* stimulation were analyzed by real-time RT-PCR. For each sample, the real-time RT-PCR analysis was performed in triplicate. A significant difference compared with the control sample at each time point (*p* < 0.05 or *p* < 0.01, *n* = 6, ANOVA) is indicated by asterisks (* or **, respectively) above the bars.

Shrimp gills and hemocytes are important tissues involved in immune defense [30]. In this study, the expression profiles of MjPellino in the gills and hemocytes after challenge with WSSV and *V. parahaemolyticus* were selected for further detection (Figure 3A–D). According to the results, the expression of MjPellino was significantly affected by these two pathogens, and the effect increased with time over a certain duration after injection. The results showed that both WSSV and one serious intracellular pathogen, *V. parahaemolyticus*, could upregulate MjPellino expression in these two tissues within a certain time period after the challenge compared with the levels detected in the sterile saline control. In the WSSV-challenged *M. japonicus* gills, MjPellino expression began to markedly increase at 24 hpi and peaked at 48 hpi (3.5-fold increase) (Figure 3A). In contrast, MjPellino expression in the hemocytes of *M. japonicus* increased gradually from 6 to 72 hpi, peaked at 72 hpi (1.7-fold increase), and was downregulated at 96 hpi (0.7-fold decrease) after the WSSV challenge (Figure 3B). In response to *V. parahaemolyticus*, MjPellino expression in the gills abruptly increased until reaching a peak of 3.2-fold at 3 hpi and showed periodic expression (Figure 3C), and similar findings were obtained in hemocytes (Figure 3D). The results showed that the levels and changes in MjPellino mRNA expression in *M. japonicus* were similar to those obtained for Pellino mRNA expression in *Litopenaeus vannamei* after challenge with *V. parahaemolyticus* [31]. The control group, which was injected with sterile saline, did not show any obvious change in MjPellino expression.

### 2.5. Three-Dimensional Modeling and Protein-Protein Docking Assays

**Figure 4:**
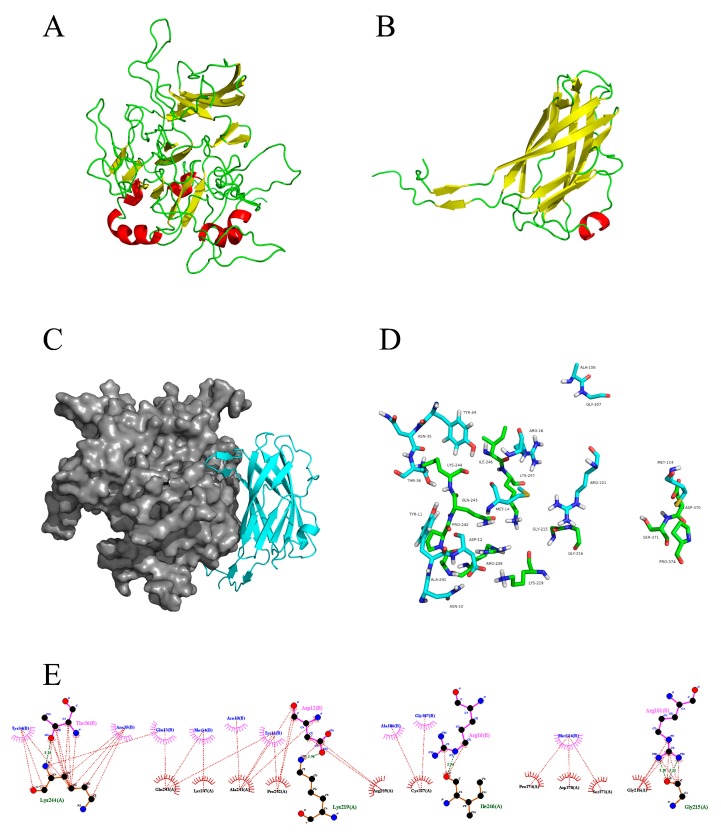
Protein interaction complexes of MjPellino with VP26 of WSSV. Three-dimensional models of (**A**) MjPellino and (**B**) VP26 of WSSV. (**C**) The three-dimensional models and interaction of MjPellino and VP26 were analyzed using Modeler 9.20 and ZDOCK. The interface residues of the MjPellino and VP26 proteins are shown as gray and blue spheres, respectively. (**D**) Action pattern diagram of MjPellino (green) and VP26 (blue). (**E**) Map of specific interaction sites and forces.

The results showed that the MjPellino and WSSV VP26 proteins could anchor to each other, and the predicted structure is shown in Figure 4A,B. The PDB [32] codes for the structural templates identified for MjPellino and VP26 were 3EGB (homologous similarity, 63.18%) and 2EDM (homologous similarity, 100%), respectively. After homology modeling and energy minimization (Supplementary Figures S3–S6), the allowable range in the full conformation diagram of MjPellino was 98.6%, which exceeded the 95% limit and meets the requirements for subsequent molecular docking or molecular dynamics models.

Fourteen residues of MjPellino (GLY215, GLY216, LYS219, ARG239, ALA241, PRO242, GLN243, LYS244, ILE246, LYS247, CYS327, ASP370, SER371, and PRO374) and 12 residues of VP26 (ASN10, TYR11, ASP12, GLN13, MET14, ARG16, TYR34, ASN35, THR36, ARG101, ALA106, and GLY107) are involved in the interaction between the two proteins (Figure 4C). The interaction forces between MjPellino and VP26 consist of hydrophobic, van der Waals, hydrogen bond, and electrostatic interactions. Among these interactions, the THR36 residue of VP26 forms a 3.25 Å-long hydrogen bond with LYS244 of MjPellino, the ARG12 residue of VP26 forms a 2.96 Å-long hydrogen bond with LYS219 of MjPellino, the ARG16 residue of MjPellino forms a 2.94 Å-long hydrogen bond with ILE216 of VP26, and the ARG101 residue of MjPellino forms hydrogen bonds with GLY215 of VP26 with bond lengths of 2.2 and 3.1 Å (Figure 4D,E). The formation of these hydrogen bonds increases the ability of the two proteins to target each other and thereby enables activation of the protein signaling pathway.

### 2.6. Expression and Purification of Recombinant Proteins

**Figure 5:**
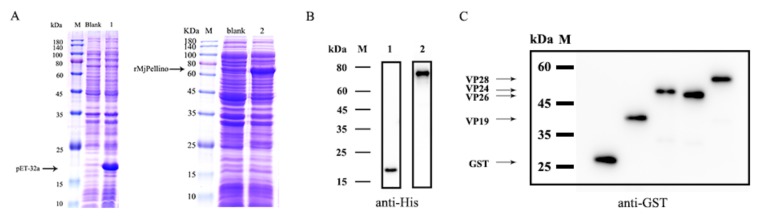
SDS-PAGE analysis of the induction of His-pET32a and rMjPellino protein expression and Western blot analysis of purified His-pET32a, rMjPellino, rVP19, rVP24, rVP26, and rVP28 proteins. (**A**) His-pET32a protein and rMjPellino expression. M, protein molecular standard; blank, total cellular extract from *E. coli* BL21(DE3) pLysS chemically competent cells before IPTG induction; 1, SDS-PAGE of pET-32a after IPTG induction; 2, SDS-PAGE of rMjPellino after IPTG induction. (**B**) Western blot analysis of pET-32a (lane 1) and rMjPellino (lane 2). (**C**) Western blot analysis of pGEX-4T-1, rVP19, rVP24, rVP26, and rVP28 (from left to right).

SDS-PAGE with Coomassie brilliant blue staining was performed to detect the expression of His-pET32a protein and the recombinant MjPellino protein (rMjPellino), and the results showed an intense band with a molecular weight of 20 kDa (His-pET32a protein) or 67 kDa (rMjPellino) (Figure 5A). A Western blotting analysis showed that the protein could bind to the His-tagged antibody (Figure 5B). The four WSSV envelope proteins rVP19, rVP24, rVP26, and rVP28 exhibited binding to the GST tag antibody (Figure 5C).

### 2.7. MjPellino Can Interact with VP26 of WSSV

**Figure 6:**
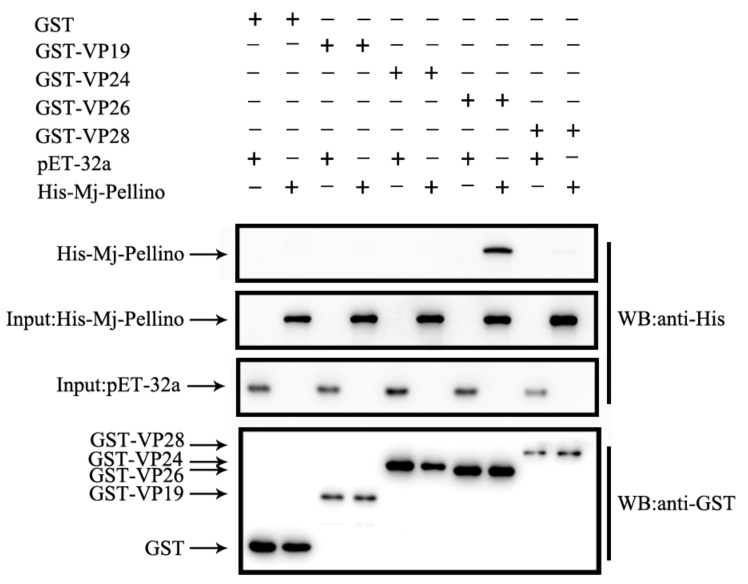
GST pull-down assay between MjPellino and four WSSV envelope proteins. The interaction of His-rMjPellino with GST-rVP19, GST-rVP26, and GST-rVP28 observed in the pull-down assay was confirmed by Western blotting with an anti-GST antibody. The symbol “+” indicates the proteins used in the GST pull-down assay.

As indicated by the qRT-PCR results, MjPellino mRNA was significantly upregulated after WSSV challenge, and the protein–protein docking assays predicted that the MjPellino and WSSV VP26 proteins could anchor to each other. A GST pull-down assay was performed to determine whether MjPellino could interact with VP26. Four important envelope proteins of WSSV were used for this detection. As shown in Figure 6, rMjPellino was able to bind to recombinant rVP26 but could not bind to rVP19, rVP24 or rVP28. The control proteins, including the His- and GST-tagged proteins, could also not bind to rVP19, rVP24, rVP26, and rVP28. The experiment demonstrated that the rMjPellino protein was able to interact with rVP26 of WSSV.

### 2.8. Inhibition of MjPellino Expression in Vivo

**Figure 7:**
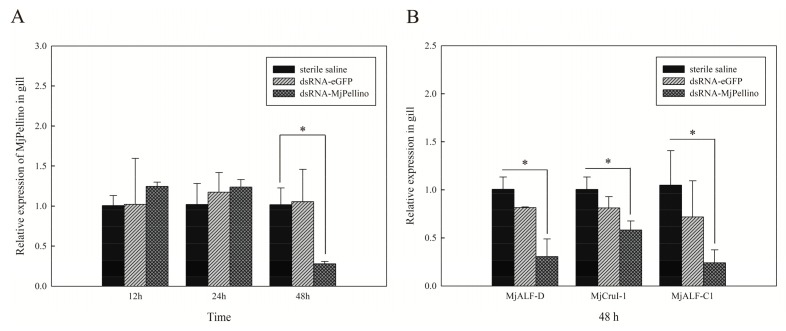
qRT-PCR analysis of MjPellino (**A**) and MjAMP (**B**) gene expression after the knockdown of MjPellino (* *p* < 0.05).

The RNAi-mediated repression of MjPellino transcription was then analyzed, and this study included an assessment of its effects on the expression of several AMPs of *M. japonicus*. MjPellino transcription in the gills was significantly suppressed (by 72%) at 48 h post-injection of dsRNA-MjPellino (*p* < 0.05), and no repression in the gills was observed in the dsRNA-eGFP group and the group injected with sterile saline (Figure 7A). The expression of three of the tested AMP genes, MjALF-D, MjALF-C1, and MjCrustin1, was downregulated 48 h after dsRNA-MjPellino injection by 69.5%, 41.8% and 76.0%, respectively, compared with the levels detected in the control group (*p* < 0.05) (Figure 7B).

## 3. Discussion

Proteins belonging to the Pellino family, which are E3 ubiquitin ligases, are emerging as important elements in tumorigenesis, innate immunity, and potentially, metabolism [33,34,35]. Pellino is one of the essential modulators of TLR signaling pathways that participate in the innate immune response [20]. In the present study, the MjPellino gene was characterized and identified from *M. japonicus* transcriptomic data. Our bioinformatic analysis suggests that MjPellino contains a RING domain and an N-terminal FHA domain. A RING-like domain with E3 ubiquitin ligase activity was previously shown to facilitate the ubiquitination of IRAK-1 [25,36], Thus, this finding indicates that MjPellino belongs to the ubiquitin ligase family. In this study, we found that the newly identified MjPellino protein is highly homologous to LvPellino, PmPellino and SpPellino; the functions of MjPellino might be similar to those of arthropod Pellino proteins, such as LvPellino and DmPellino. Previous studies with *L. vannamei* revealed that LvPellino can positively regulate the transcription factor NF-κB and that this transcription factor can activate the corresponding immune response-related genes [27]. Therefore, we speculated that the MjPellino gene might also exert an active immune regulatory effect.

In this study, the tissue distribution and time-course expression patterns of MjPellino in response to WSSV and *V. parahaemolyticus* infection were further investigated. The Pellino gene was expressed in all the tested tissues of *M. japonicus*, and its highest expression was detected in the gills; this finding was similar to that obtained for PmPellino [37], but different from that obtained with LvPellino [27]. The gills serve as the first line of defense against immune-related stress in shrimp [38]. The high expression of MjPellino in the gills might help *M. japonicus* resist external environmental stresses. Furthermore, the gills and hemocytes, which are important immune organs of shrimp, were selected as the test tissues for immune stimulation in this study. The results revealed that the mRNA expression level of MjPellino in both of these immune organs could be generally upregulated after stimulation with WSSV and *V. parahaemolyticus*, which is similar to the results obtained for LvPellino of *L. vannamei* [27]. This finding indicates that MjPellino is involved in the innate immunity of *M. japonicus*. The expression patterns of MjPellino were not completely equal in different tissues because the TLR/NF-κB pathway in invertebrates is associated with various signal transduction pathways that induce immune responses to different stimuli [31].

In shrimp, WSSV activates the Toll and IMD signaling pathways, which are two NF-κB signaling pathways [39]. The role of the mRNA expression level of Pellino is the focus of most previous studies. Researchers have confirmed that Pellino and TRAF6 can participate in Toll pathway signaling via the interaction of Pelle with Tube [27,40]. Although these studies provide important insights, the studies also have some limitations, including the effects of Pellino gene expression after WSSV challenge on the Pellino protein expression level. To further understand the mechanism underlying the recognition of WSSV by the protein encoded by the Pellino gene, we performed analyses at the protein expression level.

According to the quantitative results from the analysis of the transcription levels, we speculated that MjPellino could bind to viral envelope proteins to participate in WSSV recognition, but additional studies are needed to confirm the results. This study constitutes the first prediction and analysis of the interaction of MjPellino with an envelope protein of WSSV using bioinformatics methods. We first performed a molecular docking study to obtain more information on the function of the interaction of MjPellino with WSSV. Through three-dimensional modeling and protein–protein docking assays of the MjPellino and WSSV, VP26 proteins revealed that these proteins exhibited strong binding affinities and could anchor and interact with each other. Three-dimensional models of MjPellino, VP26, and the MjPellino-VP26 heterodimeric complex were analyzed. The coordinates of the WSSV envelope protein VP26 (PDB ID of 2EDM) were obtained from PDB [41,42]. The open interface on the surface of MjPellino is fully unfolded, and VP26 occupies the surface of MjPellino by folding at the N terminus. The three main folded lamellar layers inside VP26 could also fully fit with the interface of MjPellino through hydrophobic and other interactions. These data suggest that MjPellino might play an important role via protein–protein interactions with VP26 of WSSV.

We then constructed and expressed recombinant versions of MjPellino and four envelope proteins of WSSV and performed a GST pull-down assay to detect their interactions. In the GST pull-down assay, MjPellino was precipitated by VP26, which further confirmed that these two proteins interact. Many studies have shown that the interaction between pathogen and host proteins is key for the induction of infection and pathogenesis [43]. The infection of host cells by WSSV is a complex process during which the virus interacts with various host cell proteins [44,45]. The major strength of this study is that it provides the first demonstration that *M. japonicus* Pellino binds to WSSV by interacting with an envelope protein of WSSV, namely, VP26. VP26 was first identified as a nucleocapsid protein [46] and was also found to serve as an envelope protein of WSSV [14,47,48,49]. As demonstrated in previous studies, VP26 functions as a matrix-like linker protein between the viral envelope and nucleocapsid, which suggests that VP26 is a key factor in the linkage between the viral envelope and the nucleocapsid of the WSSV virion. This protein might be instrumental in the trafficking of the WSSV nucleocapsid into the host nucleus via the cytoskeleton [49,50,51]. Because the VP26 protein might anchor viral envelope membranes of WSSV to allow interacting with the host, we speculated that MjPellino is involved in the immune response to WSSV.

WSSV replication is induced up by the activation of the NF-κB pathway [31]; AMP genes have been reported to be crucial genes downstream of the NF-κB pathway that reduce the expression of the effectors [52]. In the present study, dsMjPellino significantly attenuated the expression of AMPs compared with that observed in the control group. These results confirm that MjPellino plays a positive role in the immune response to WSSV.

In conclusion, MjPellino of kuruma prawn, *M. japonicus*, was detected in all tested tissues, and the expression of MjPellino was upregulated in the gills and hemocytes after WSSV and *V. parahaemolyticus* challenge, which indicates that MjPellino might play a functional role in the immune response. In addition, this study provides the first demonstration that MjPellino can interact with the important envelope protein VP26 of WSSV, as shown at the protein level through the successful construction of rMjPellino. Furthermore, MjPellino can interact with the WSSV envelope protein VP26, as demonstrated through a GST pull-down assay and protein–protein docking analyses. In addition, the knockdown of MjPellino decreased the expression of MjAMP genes. Thus, MjPellino might play an important role in the immune response of *M. japonicus*. Our findings will allow further exploration and elucidation of the molecular mechanism underlying the role of the Pellino gene in the innate immune response to WSSV.

## 4. Materials and Methods

### 4.1. Animals Used for Research

All healthy *M. japonicus* (average weight 11.46 ± 1.42 g, body length 96.42 ± 2.25 mm) were procured from a large shrimp farm in Zhangzhou City, Fujian Province, China. The shrimps were domesticated in environmentally controlled tanks in 70 cm, 50 cm, and 20 cm size with 28‰ of salinity and 22–23 °C of seawater pumped with air for at least a week prior to the infection experiment. The seawater was renewed and filtered daily, and the prawns were fed twice a day with commercial feed (Fuxing (Xiamen, China) organism feed co. LTD, at least 45% crude protein content). All experimental procedures involving animals were carried out in accordance with the Regulations for the Administration of Affairs Concerning Experimental Animals, approved by the State Council on 31 October 1988 and promulgated by Decree No.2 of State Science and Technology Commission on 14 November 1988.

### 4.2. Cloning of the Full-Length Mjpellino Gene

Total RNA from the gills and hepatopancreas of healthy *M. japonicus* was obtained using the TRIzol reagent (Invitrogen, Carlsbad, CA, USA). cDNA synthesis was performed using the PrimeScript 1^st^ Strand cDNA Synthesis Kit (Takara, Kyoto, Japan) with oligo d(T), random hexamer reverse transcription primers and specific primers according to the manufacturer′s instructions. The product was stored at −20 °C until subsequent use. Rapid amplification of cDNA ends (RACE) was performed using the SMARTer™ RACE 5′/3′ Kit (Clontech, Shiga, Japan) according to the user′s manual to obtain the full-length sequence. All primer sequences are provided in Table 1. Using Primer Premier 5.0 software, the primers were designed based on a partial sequence predicted to encode a Pellino homolog protein retrieved from the *M. japonicus* transcriptome library analyzed by our laboratory [53]. The final PCR products were cloned into the pMD-19T vector (Takara, Kyoto, Japan), and positive clones were selected and sequenced. The sequencing results were identified using the BLAST online program [54]. The full-length cDNA of MjPellino was finally obtained by splicing the mid-fragment sequence and 5′- and 3′-end sequences.

### 4.3. Immune Challenge Assays and Tissue Collection

For MjPellino gene expression analyses, the second abdominal segment of each *M. japonicus* was injected with 50 µL of sterile saline (0.9% NaCl) containing WSSV (10^6^ copies) or *V. parahaemolyticus* (2 × 10^5^ CFU/mL) as the immune source or 50 µL of sterile saline as the negative control using a 1-mL medical insulin syringe. The hemocytes and gills were sampled 3, 6, 12, 24, 48, 72, and 96 h post injection. To detect the tissue distribution of MjPellino mRNA, various tissues (gill, hepatopancreas, heart, intestine, stomach, muscle, eyestalks, and hemocytes) were sampled from six untreated healthy *M. japonicus.* Hemocytes were collected by puncturing the heart of each prawn with a sterile syringe preloaded with anticoagulant, and the resulting sample was centrifuged at 5000 rpm and 4 °C for 8 min. Subsequently, 1 mL of TRIzol reagent was added to each centrifuge tube, and the samples were stored at −80 °C until further analysis. All other dissected tissues were placed in RNAfixer (Aidlab Biotechnologies, Beijing, China) immediately, stored overnight at 4 °C and then transferred to −20 °C.

### 4.4. Quantitative Real-Time PCR Analysis

Through quantitative real-time PCR (qRT-PCR), the expression level of MjPellino mRNA was measured using an Applied Biosystems 7500 Fast real-time PCR system (Applied Biosystems, Foster City, CA, USA). RNA extraction was performed as described above in Section 4.2, and the RNA was subsequently reverse transcribed to cDNA by TB Green ^®^ Premix DimerEraser™ (Perfect Real Time) (Takara, Kyoto, Japan) based on the manufacturer′s protocol, and Table 1 shows the primers used in this section. Primers designed for EF1-α were used as the internal reference for standardization [55]. A volume of 20 µL RT-PCR reaction mixture included TB Green Premix DimerEraser (2×) 10 µL, each primer (10 mM) 0.6 µL, Rox Reference Dye II (50×) 0.4 µL, cDNA 2 µL (diluted 1:10 in EASY Dilution for real time PCR), and RNase-free water 6.4 µL. The cycle conditions were described as follows: 95 °C for 30 s for 1 cycle, 40 cycles of 95 °C for 5 s, 59 °C for 30 s, and 72 °C for 30 s, and the melting curve analysis was implemented followed by the thermocycling conditions to confirm the specificity of the amplification and ensure successful amplification and detection. The expression levels of MjPellino were quantified using the 2^−ΔΔ*C*t^ method after normalization to EF-1α [56]. Triplicate reactions of each sample were performed, and all the primer sequences are listed in Table 1.

### 4.5. Three-Dimensional Modeling and Protein-Protein Docking Assays

Most biological research involves the functional characterization of protein sequences [57]. Molecular docking, an important tool in structural molecular biology, can predict the predominant binding mode of a ligand with a protein with a known 3D structure [58]. To further explore the reason why MjPellino can respond to WSSV infection, the potential interactions and the binding sites of MjPellino with envelope proteins of WSSV were studied using a molecular docking approach. Three-dimensional models of these proteins were generated using Protein Data Bank (PDB) (https://www.rcsb.org/) and Modeler 9.20 software. To ensure the quality of the model, multi-template modeling was selected for protein construction. The lowest rated conformation of DOPE was selected, protein structural energy minimization was performed using GROMACS 5.1.4 software, and Ramachandran plots were prepared [59] to judge the quality of the protein structures and thus confirm the final protein model. The molecular docking analyses between MjPellino and VP26 were performed using ZDOCK [60], which is a protein-docking program based on fast Fourier transform provided and maintained by ZLAB, University of Massachusetts Medical School [61]. The ZDOCK program is used for searching all possible binding patterns between two proteins in the space created by translation and rotation, and through energy-based scoring function to assess each binding model [62]. After the conformation was docked 100 times, the lowest energy conformation was selected as the final result.

### 4.6. Construction of the Recombinant Prokaryotic Plasmid and Protein Expression

To further study the function of MjPellino, we constructed recombinant prokaryotic plasmids harboring MjPellino and four WSSV membrane proteins, namely, VP19, VP24, VP26, and VP28. In brief, we designed primers using Primer Premier 5 software according to the sequences listed in Table 1. The entire coding region of MjPellino was searched for BamHI/HindIII restriction sites and then subcloned into the expression vector pET-32a with T4 DNA ligase (Takara, Kyoto, Japan). The four WSSV structural genes VP19, VP24, VP26, and VP28 were cloned into the pGEX-4T-1 plasmid with specific primers. After identification by sequencing and restriction endonuclease treatment, all the recombinant plasmids were transferred into *E. coli* BL21 (DE3) pLysS chemically competent cells (Transen Biotech, Beijing, China). Positive clones harboring the desired fragment were then selected for the induction of expression. The IPTG concentration and the temperature used for induction were 0.1 mM and 18 °C, respectively. The clone was inoculated into fresh LB medium and cultured at 37 °C until the final OD600 reached approximately 0.6–0.7. Subsequently, 0.1 mM IPTG was added to the medium, and the temperature was maintained at 18 °C for 12 h to induce expression. On the second day, the induced bacteria were collected, ultrasonicated and centrifuged for isolation of the supernatants and sediments. The supernatants were purified using HisCap Smart 6FF (Smart-lifesciences, Changzhou, China) with an AKTA Purifier 100 (GE Healthcare Life Sciences, Pittsburgh, PA, America). The rVP19, rVP24, rVP26, and rVP28 fused proteins were purified using a *ProteinIso*^®^ GST resin affinity chromatographic column (TransGen Biotech, Beijing, China). The expression plasmid pET-32a-MjPellino-His and expression plasmids harboring the four major WSSV envelope proteins (VP19, VP24, VP26, and VP28) with a GST-tag were constructed. The N-terminal hydrophobic regions of the VP26 protein were removed to ensure soluble expression. The purified rMjPellino, rVP19, rVP24, rVP26, and rVP28 were used for Western blot analysis.

### 4.7. GST Pull-Down and Western Blot Analysis

To further explore and confirm this hypothesis, whether MjPellino can interact with the four most important WSSV membrane proteins, namely, VP19, VP24, VP26 and VP28a, was determined through GST pull-down assays. The four purified fusion proteins (GST-VP19, GST-VP24, GST-VP26, and GST-VP28) were combined with the pGEX-4T-1 vector, which was used as the bait protein, namely, the pGEX-4T-1 no-load protein (GST), the purified pET-32a-MjPellino fusion protein (His-MjPellino) and His-pET32a no-load protein (His-pET32a) were used as the prey proteins. In the GST pull-down assay, the glutathione Sepharose beads were washed eight times with 1× PBS to release the alcohol protector, and 10 mL of GST, GST-VP19, GST-VP24, GST-VP26 or GST-VP28 was then added. The beads were shaken at room temperature for 2 h. After removing the supernatant, the glutathione Sepharose beads were washed eight times with 1× PBS (with 1‰ Triton X-100). His-pET32a or His-MjPellino was added to the glutathione Sepharose beads, and the mixture was incubated overnight at 4 °C with rotation and washed eight times with 1× PBS (with 1‰ Triton X-100) to remove unbound proteins. Subsequently, the immobilized proteins on the beads were separated by SDS-PAGE, transferred to a PVDF membrane (voltage: 100 V, time: 1 h) and analyzed by Western blotting. The membranes were subsequently subjected to three 10-min washes with PBST. Using 5% skimmed milk as the blocking solution, the membranes were blocked overnight at 4 °C, incubated with ProteinFind anti-His mouse monoclonal antibody (TransGen Biotech, Beijing, China) (diluted 1:1000) or ProteinFind anti-GST mouse monoclonal antibody for 2 h at 4 °C, and immersed in ProteinFind goat anti-rabbit IgG(H+L) (HRP) antibody (TransGen Biotech, Beijing, China) (diluted 1:2000) for 2–3 h at room temperature. The membranes were screened with the *EasySee*^®^ Western Blot Kit (TransGen Biotech, Beijing, China) according to the manufacturer′s instructions, and images were obtained.

### 4.8. Knockdown of MjPellino and Analysis of Its Effect on MjAMP Gene Expression

T7 RNA polymerase was used for in vitro transcription to synthesize dsMjPellino and dseGFP, which were verified by sequencing using an in vitro Transcription T7 Kit (for siRNA Synthesis) (TaKaRa, Japan) according to the recommended instructions. The primers are shown in Table 1. For the RNAi experiment, 48 shrimps (10.96 ± 1.12 g) were divided into three equal groups which were intramuscularly injected with 50 μL of dsMjPellino (the experimental group), dseGFP (negative control group) and saline (blank control group), respectively. At 12, 24 and 48 h post injection, gills were obtained from five randomly shrimp in each group for analyzing the expression of MjPellino using qRT-PCR as described in Section 4.4. Then, the expression levels of MjAMP genes in the gills at 48 hpi were analyzed. The MjAMP genes included MjALF-D (anti-lipopolysaccharide factor gene segment from the *M. japonicus* transcriptome), MjALF-C1 (GenBank accession number AB210110), and MjCruI-1 (GenBank accession number KU160502). The primer sequences are listed in Table 1.

### 4.9. Bioinformatics Analysis and Statistical Analysis

The ORFs of the cDNA and the deduced AAs of MjPellino were predicted using EditSeq software from DNAStar. BLAST online software [54] was used to compare the obtained MjPellino sequences to the sequence base. The functional domains of the proteins were predicted using the Simple Modular Architecture Research Tool (SMART) [63]. ExPASy online software [64] was used to predict the protein pI and molecular mass. All of the protein sequences of Pellino homologs from other species were downloaded from NCBI. Multiple-sequence alignments were performed using Clustalx1.83 and the multiple-sequence alignment display program ESPript3.0 [53,65,66,67]. A neighbor-joining (NJ) phylogenetic tree was constructed with 1000 bootstrap replications using MEGA7.0 software.

The relative mRNA levels in different tissues and different treatment groups were compared by one-way analysis of variance (ANOVA) followed by multiple comparison testing with Student’s test using SPSS PASW statistical analysis software. All the data are provided in terms of the mean relative mRNA expression levels ± standard deviations of the means (SDs). Differences were considered significant if *p* < 0.05 and highly significant if *p* < 0.01.

## Figures and Tables

**Table 1:** Primers used in this study.

Primer	Sequence (5′–3′)	Sequence Information
Pellino-5′-out	AGGTGTTTCAGACGACCGACCAATTTGA	5′-RACE PCR
Pellino-5′-in	CCACAATCACCGCTTGGTTTCGTGACAGT	5′-RACE PCR
Pellino-3′-out	CAGGTGAAACTCAATTTGTCAAGACATAT	3′-RACE PCR
Pellino-3′-in	AAGTAGAGTGAATCAAACCCACTGCTGGG	3′-RACE PCR
M13-F	TGTAAAACGACGGCCAGT	PCR
M13-R	CAGGAAACAGCTATGACC	PCR
EF1-α-F	GGAACTGGAGGCAGGACC	internal control
EF1-α-R	AGCCACCGTTTGCTTCAT	internal control
Pellino-qPCR-F	TGGAGTATCAGTTAGACACCGAGACA	qRT-PCR
Pellino-qPCR-R	GCGAGTTGTTCTGTGAGCAATTATACG	qRT-PCR
T7-F	TAATACGACTCACTATAGG	recombinant protein
T7-R	TGCTAGTTATTGCTCAGCGG	recombinant protein
Pellino-BamHI-F	CGCGGATCCGCGATGCCCGACCCTCCTATAAT	recombinant protein
Pellino-HindIII-R	CCCAAGCTTGGGTCAGTCGCAGTTGTCTTGAAAAATGAGTC	recombinant protein
VP19-F	CGCGGATCCATGGCCACCACGACTAACAC	recombinant protein
VP19-R	CCGGAATTCTTATCCCTGGTCCTGTTCTTATATT	recombinant protein
VP24-F	CGCGGATCCAACATAGAACTTAACAAGAAATTGGAC	recombinant protein
VP24-R	CCGGAATTCTTATTTTTCCCCAACCTTAAACA	recombinant protein
VP26-F	CGCGGATCCACACGTGTTGGAAGAAGCGT	recombinant protein
VP26-R	CCGGAATTCTTACTTCTTCTTGATTTCGTCCTTG	recombinant protein
VP28-F	CGCGGATCCATGGATCTTTCTTTCACTCTTTCG	recombinant protein
VP28-R	CCGGAATTCTTACTCGGTCTCAGTGCCAGAG	recombinant protein
dsRNA-MjPellino-T7-F	GATCACTAATACGACTCACTATAGGGAAGCGGTGATTGTGGAGTATC	RNAi
dsRNA-MjPellino-T7-R	GATCACTAATACGACTCACTATAGGGCATAGTAACGTAGCTCCACACAAGTC	RNAi
dseGFP-T7-F	GATCACTAATACGACTCACTATAGGGACCCTCGTGACCACCCTGAC	RNAi
dseGFP-T7-R	GATCACTAATACGACTCACTATAGGGTCTCGTTGGGGTCTTTGCTC	RNAi
MjALF-D-qPCR-F	CTTCCTCCTCAGTGACCAGTCCT	qRT-PCR
MjALF-D-qPCR-R	AGAATCCGAAACTCGCAGCCAAT	qRT-PCR

## Supplementary Materials

The following are available online at https://www.mdpi.com/1422-0067/21/4/1243/s1, Figure S1: Multiple-sequence alignments of the deduced AA sequences of MjPellino with other known Pellino sequences; Figure S2: Phylogenetic analysis of Pellino proteins from various animals; Figure S3: Energy minimization potential energy diagram of MjPellino (Gromacs); Figure S4: Ramachandran plot of MjPellino; Figure S5: Energy minimization potential energy diagram of VP26 of WSSV (Gromacs); Figure S6: Ramachandran plot of VP26 of WSSV.

## Abbreviations

MDPI	Multidisciplinary Digital Publishing Institute
DOAJ	Directory of open access journals
TLA	Three letter acronym
LD	linear dichroism
EF1-α	elongation factor 1-α
ZLAB	Zhiping Weng′s laboratory
eGFP	enhanced green fluorescent protein
