# Supplementary material for: Identification and Molecular Characterization of a Pellino Protein in Kuruma Prawn (Marsupenaeus Japonicus) in Response to White Spot Syndrome Virus and Vibrio Parahaemolyticus Infection
Source: Int J Mol Sci. 2020 Feb 13;21(4):1243. doi: 10.3390/ijms21041243 (PMC7072872; doi:10.3390/ijms21041243)
Supplement: Supplementary file 1 [file ijms-21-01243-s001.pdf]

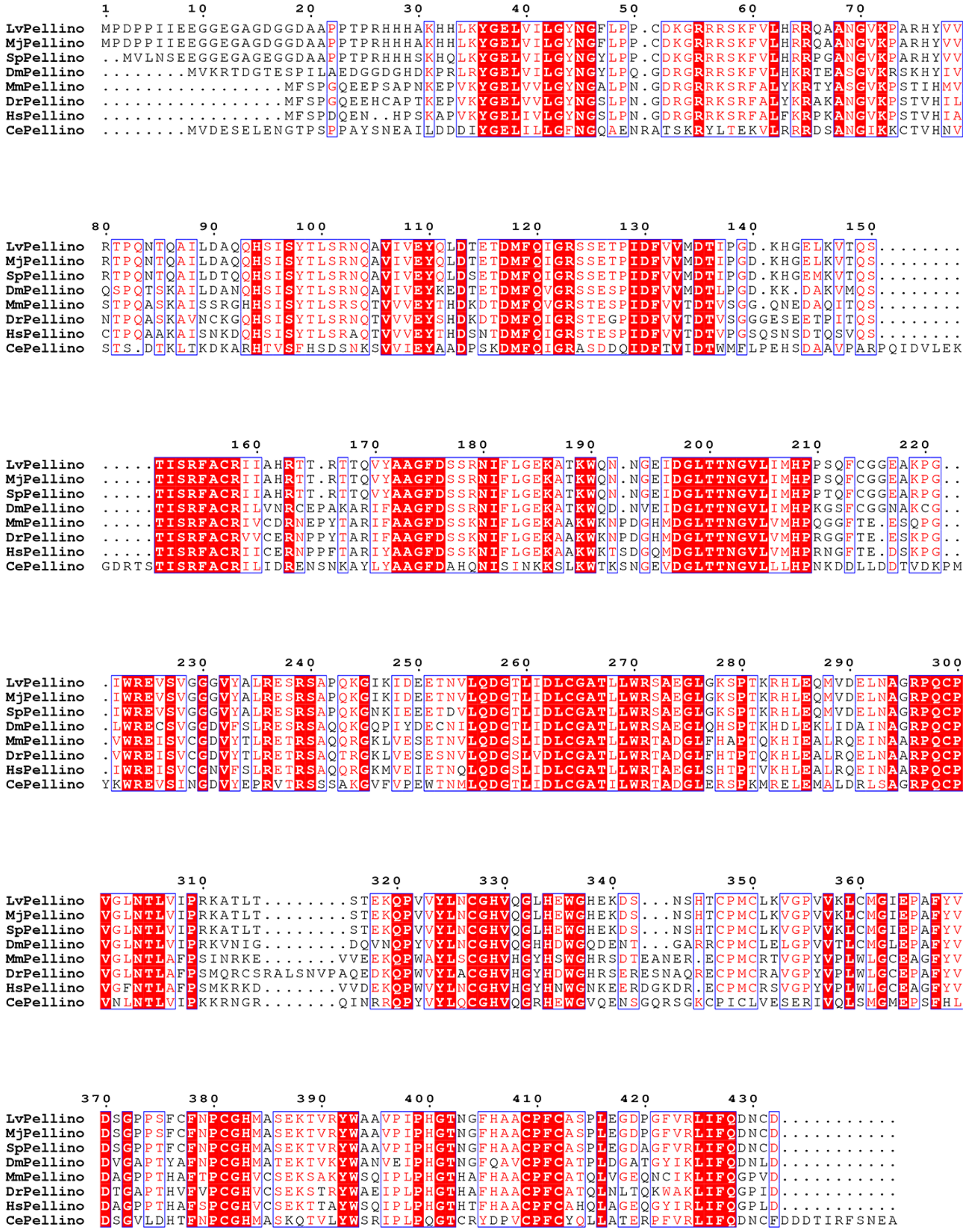

Figure S1. Multiple-sequence alignments of the deduced AA sequences of MjPellino with other known Pellino sequences. Highly conserved AA residues are shown within blue boxes, and identical residues are shaded in red. The GenBank accession numbers of the sequences are as follows: *Caenorhabditis elegans* Pellino (accession no. CAB97346.1), *Drosophila melanogaster* Pellino (accession no. NP_524466.1); *Danio rerio* Pellino (accession no. NP_001002616.1), *Homo sapiens* Pellino (accession no. NP_065702.2), *Mus musculus* Pellino (accession no. NP_075813.2), *Scylla paramamosain* Pellino (accession no. SAY41342.1) and *Litopenaeus vannamei* Pellino (accession no. KC346863.1).

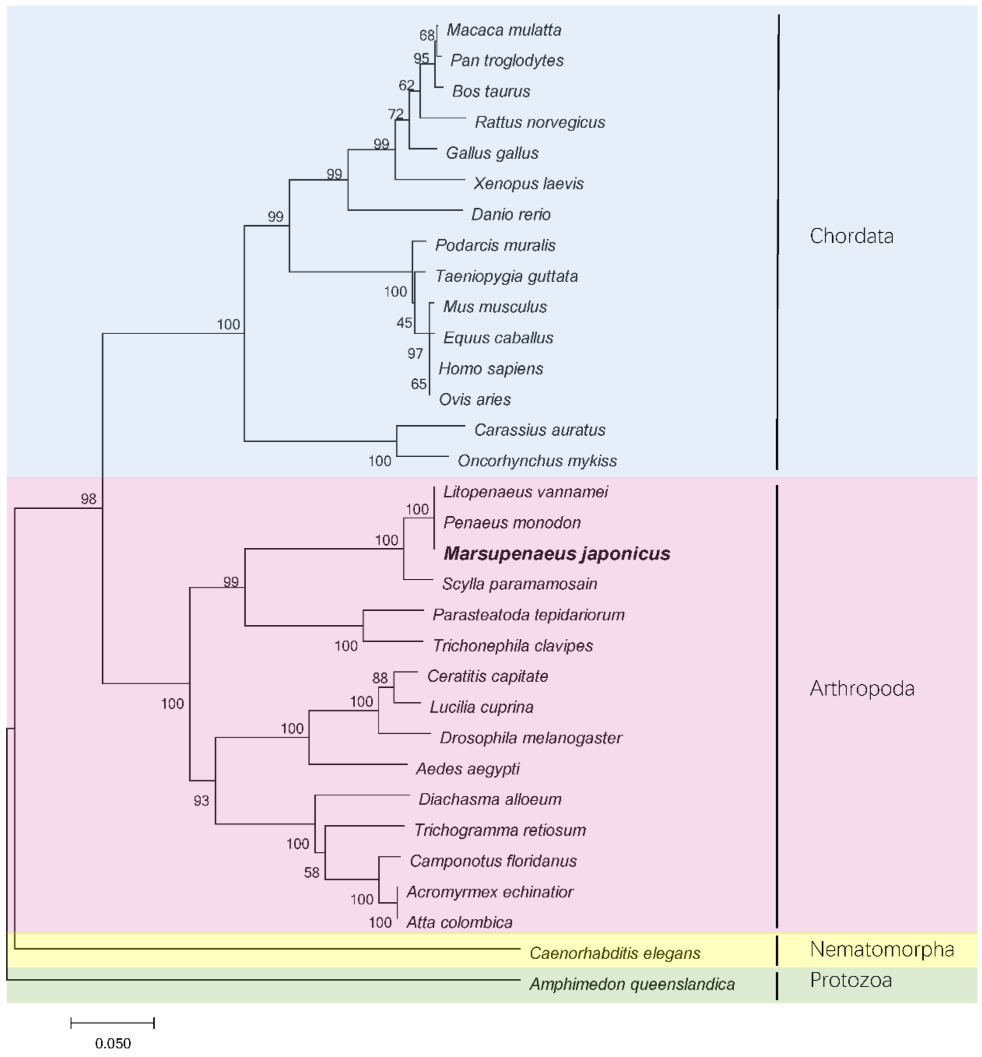

Figure S2. Phylogenetic analysis of Pellino proteins from various animals. MjPellino is marked in large bold font. MEGA 7.0 software was used based on the deduced AA sequence alignments obtained with ClustalW. The reproducibility of the results was examined with 1000 bootstraps. The proteins included in the analysis are the following: *Macaca mulatta* (accession no. XP_014999255.1), *Pan troglodytes* (accession no. XP_522860.2), *Bos taurus* (accession no. XP_002690998.1), *Rattus norvegicus* (accession no. NP_001100729.1), *Gallus galls* (accession no. XP_001234988.1), *Xenopus laevis* (accession no. NP_001085528.1), *Danio rerio* (accession no. NP_001002616.1), *Podarcis muralis* (accession no. XP_028577713.1), *Taeniopygia guttata* (accession no. XP_002199682.1), *Mus musculus* (accession no. NP_075813.2), *Equus caballus* (accession no. XP_001493996.1), *Homo sapiens* (accession no. NP_065702.2), *Ovis aries* (accession no. XP_012023818.1), *Carassius auratus* (accession no. XP_026122389.1), *Oncorhynchus mykiss* (accession no. XP_021471009.1), *Litopenaeus vannamei* (accession no. KC346863.1), *Penaeus monodon* (accession no. QBY06350.1), *Scylla paramamosain* (accession no. SAY41342.1), *Parasteatoda tepidariorum* (accession no. XP_015916048.1), *Trichonephila clavipes* (accession no. PRD24637.1), *Ceratitis capitata* (accession no. XP_023158998.1), *Lucilia cuprina* (accession no. XP_023308808.1), *Drosophila melanogaster* (accession no. NP_524466.1), *Aedes aegypti* (accession no. XP_021709421.1), *Diachasma alloeum* (accession no. XP_015125785.1), *Trichogramma pretiosum* (accession no. XP_014229022.1), *Camponotus floridanus* (accession no. EFN68970.1), *Acromyrmex echinatior* (accession no. EGI69887.1), *Atta colombica* (accession no. KYM75401.1), *Caenorhabditis elegans* (accession no. CAB97346.1), and *Amphimedon queenslandica* (accession no. NP_001266222.1).

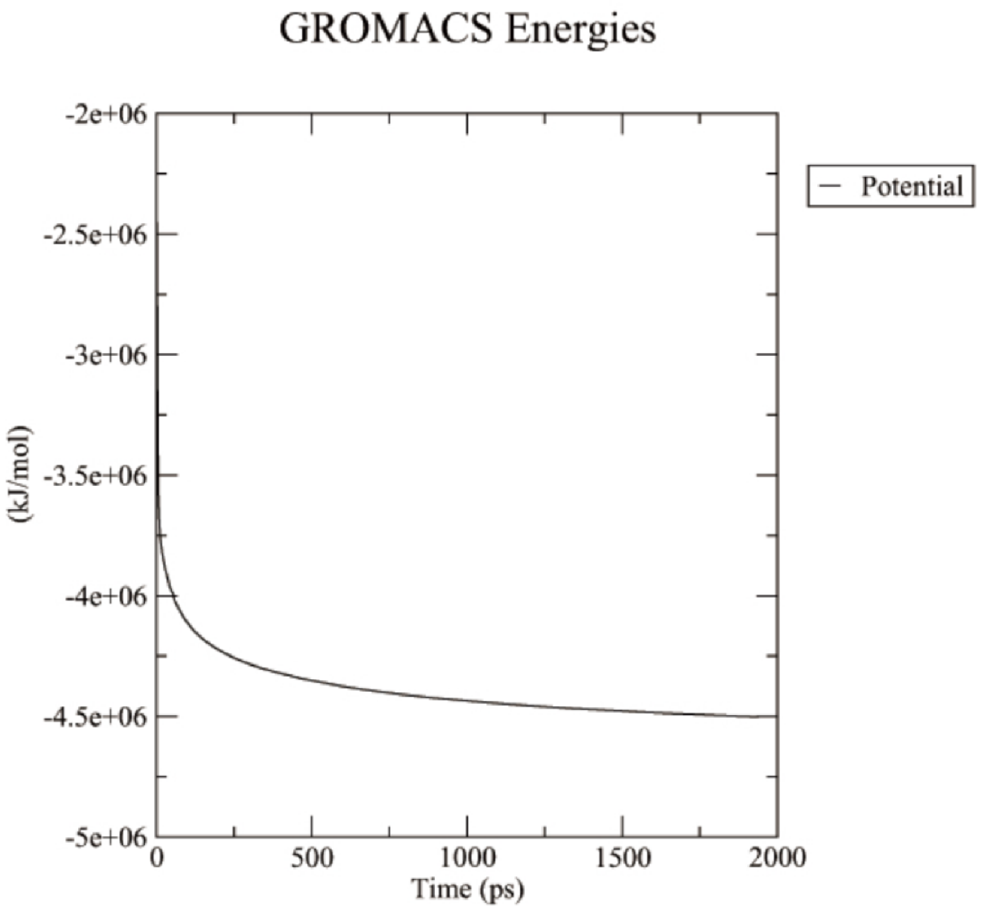

Figure S3. Energy minimization potential energy diagram of MjPellino (Gromacs)

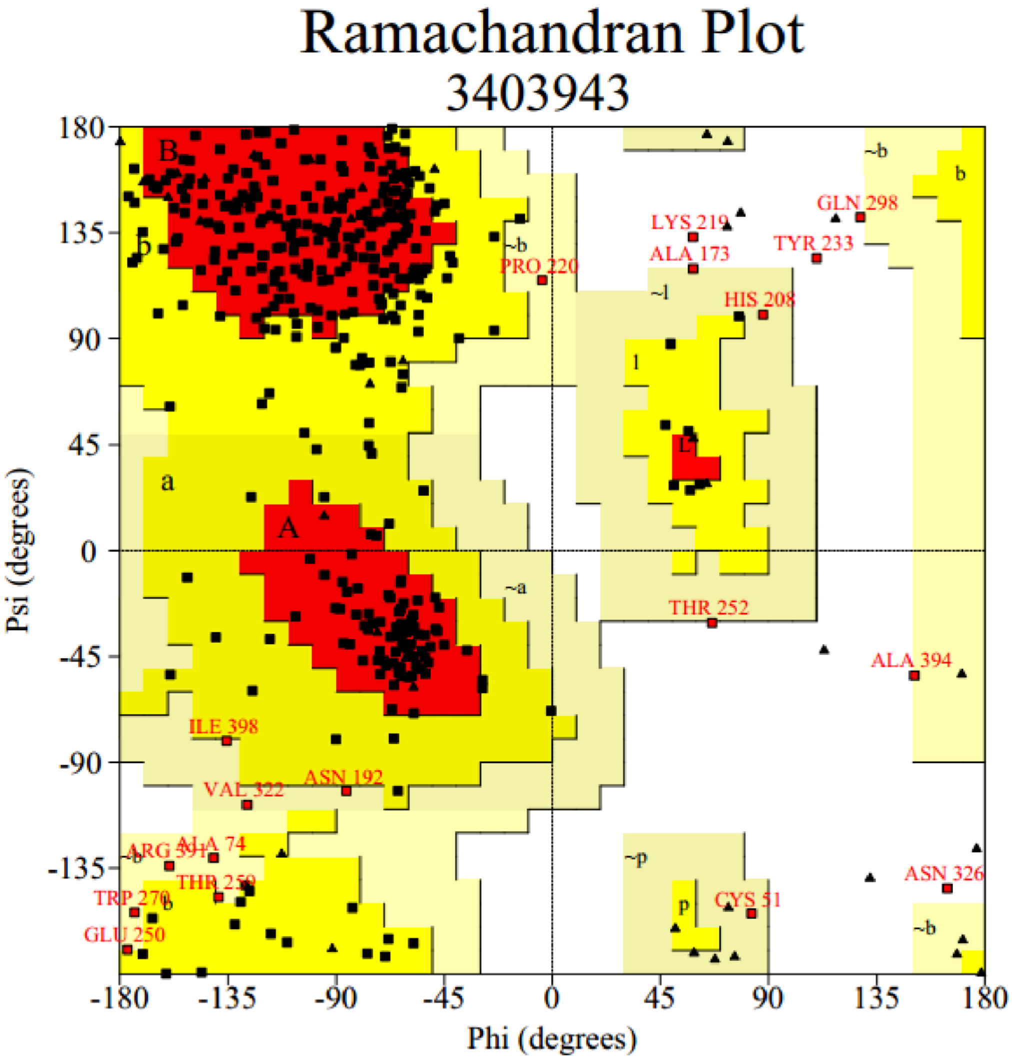

Plot statistics

Residues in most favoured regions [A,B,L]	244	68.7%
Residues in additional allowed regions [a,b,l,p]	94	26.5%
Residues in generously allowed regions [~a,~b,~l,~p]	12	3.4%
Residues in disallowed regions	5	1.4%
	----	------
Number of non-glycine and non-proline residues	355	100.0%
Number of end-residues (excl. Gly and Pro)	2	
Number of glycine residues (shown as triangles)	43	
Number of proline residues	32	

Total number of residues	432	

Figure S4. Ramachandran plot of MjPellino

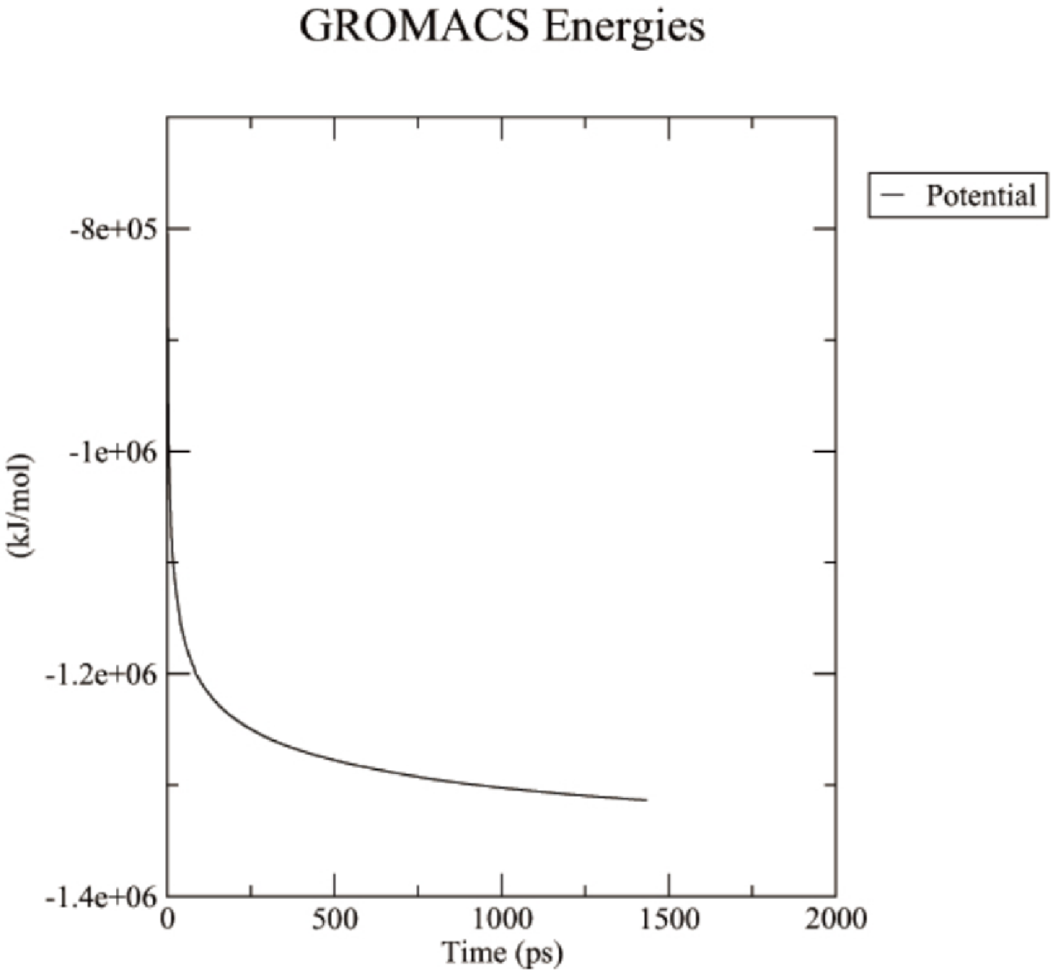

Figure S5. Energy minimization potential energy diagram of VP26 of WSSV (Gromacs)

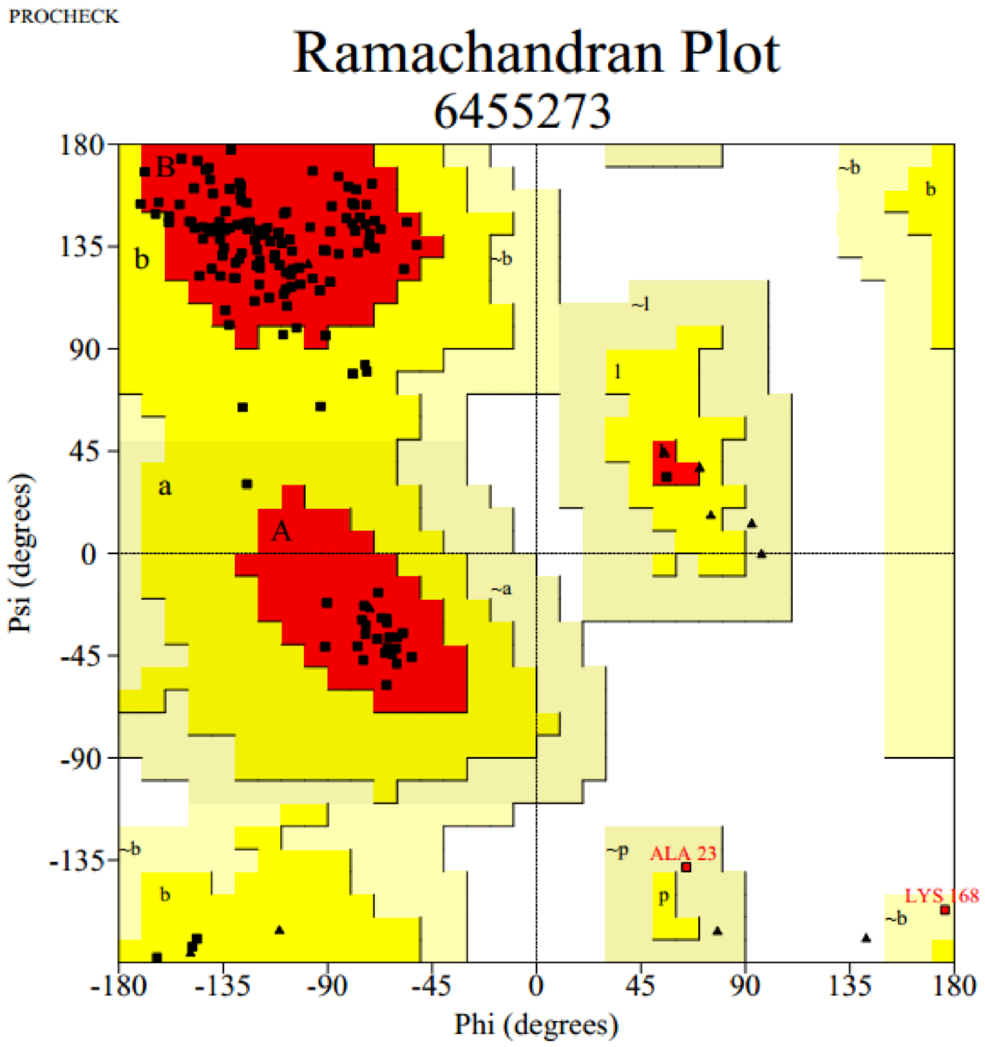

Plot statistics

Residues in most favoured regions [A,B,L]	137	90.7%
Residues in additional allowed regions [a,b,l,p]	12	7.9%
Residues in generously allowed regions [~a,~b,~l,~p]	2	1.3%
Residues in disallowed regions	0	0.0%
	----	------
Number of non-glycine and non-proline residues	151	100.0%
Number of end-residues (excl. Gly and Pro)	2	
Number of glycine residues (shown as triangles)	11	
Number of proline residues	5	

Figure S6. Ramachandran plot of VP26 of WSSV
